# Mapping of urban garden soil microbiomes in Bangladesh

**DOI:** 10.1128/mra.00375-25

**Published:** 2025-06-09

**Authors:** M. Nazmul Hoque, Md. Liton Rana, Md. Abu Ahsan Gilman, Pritom Kumar Pramanik, Md. Saiful Islam, Sadia Afrin Punom, Jayedul Hassan, Tofazzal Islam, Srinivasan Ramasamy, Pepijn Schreinemachers, Ricardo Oliva, Md. Tanvir Rahman

**Affiliations:** 1 Molecular Biology and Bioinformatics Laboratory, Department of Gynecology, Obstetrics and Reproductive Health, Gazipur Agricultural University, Gazipur, Bangladesh; 2 Department of Microbiology and Hygiene, Faculty of Veterinary Science, Bangladesh Agricultural University 54492, Mymensingh, Bangladesh; 3 National Engineering Research Center of Industrial Wastewater Detoxication and Resource Recovery, Research Center for Eco-Environmental Sciences, Chinese Academy of Sciences, Beijing, China; 4 University of Chinese Academy of Sciences 74519, Beijing, China; 5 Department of Animal Sciences, University of California-Davis 8789, Davis, California, USA; 6 Institute Biotechnology and Genetic Engineering, Gazipur Agricultural University, Gazipur, Bangladesh; 7 World Vegetable Center 70333, Shanhua, Tainan, Taiwan; 8 World Vegetable Center, Bangkok, Thailand; DOE Joint Genome Institute, Berkeley, California, USA

**Keywords:** soil, rooftop, surface, garden, microbiomes, urban agriculture

## Abstract

Shotgun metagenomics revealed distinct microbiome profiles in the garden soils of Dhaka and Gazipur district, Bangladesh, with *Bacillus* spp. demonstrating ecological dominance (>53% relative abundance) and location-specific distribution patterns. These findings highlight *Bacillus* species as prevalent microbes in urban garden soils.

## ANNOUNCEMENT

Urbanization alters land use and soil conditions, influencing microbial diversity and ecosystem functions (1–3). Soil microorganisms are essential for nutrient cycling and plant health but are increasingly threatened by urban pollution, waste, and farming practices, which may also contribute to the spread of antibiotic resistance genes (4, 5). Among these microbes, *Bacillus* species are widespread and functionally important, supporting plant resilience and soil fertility (6). As urban agriculture expands, especially in densely populated areas, understanding soil microbial communities becomes critical. This study investigates the microbial diversity and community structure of rooftop and surface garden soils in Dhaka and Gazipur using whole metagenome shotgun (WMS) sequencing.

This study analyzed 27 soil samples collected from rooftop and surface gardens in Dhaka and Gazipur districts, Bangladesh (Table 1), comprising six Dhaka rooftop garden (DRG), six Dhaka surface garden (DSG), eight Gazipur rooftop garden (GRG), and six Gazipur surface garden (GSG). These gardens, located 15 km–20 km apart (23.72–23.99°N, 90.40-90.42°E), experience a tropical monsoon climate with 1,875 mm average rainfall, 26.1°C mean annual temperature, and 65.8% humidity (3). Composite soil samples (0 cm–5 cm depth) were aseptically collected during April–July 2024 using 2.5 cm diameter hand trowels (3), homogenized, sieved (6.35 mm), and split into two 1.0 g technical replicates. DNA was extracted using the DNeasy PowerSoil Kit (QIAGEN, Germany) (7) and quantified with NanoDrop ND-2000 (4). Libraries were prepared with Nextera XT (4, 7, 8), assessed via Qubit 2.0 and Agilent Bioanalyzer 2100, and sequenced (2 × 150 bp) on Illumina NovaSeq 6000 by Macrogen Inc. (South Korea). WMS reads were processed using Trimmomatic v.0.39 (9) and BBDuk incorporated in BBTools (v.32.32) (8), then analyzed via CZ ID pipeline (v.7.1) (10). CZ ID pipeline is a cloud-based workflow that maps reads to the NCBI (nucleotide) and NR (non-redundant protein) databases using STAR for host filtering (optional), GSNAPL for nucleotide alignment, and Rapsearch2 for protein alignment. Phyloseq R package, v.4.4 (11), was used for data processing and subsequent taxonomic analysis. Statistical comparisons employed Kruskal-Wallis tests and SPSS v25.0 (12). All tools were run with default parameters unless otherwise specified.

**TABLE 1:** Study sample information, SRA accession numbers of the whole metagenome shotgun sequences, and OTUs (operational taxonomic units = 766) mapped against microbial taxa^*a*^

Sl. no.	Sample ID	Collection site	Coordinates	Source	No. of raw reads (before trimming)	No. of quality reads (after trimming)	No. of mapped reads	GC content (%)	No. of observed OTUs	SRA accessions
1	DR1	DNCC, Bangladesh	23.78° N, 90.41° E	DRG	59,967,810	59,559,296	1,393,674	39.46	180	SRR32835637
2	DR2	DNCC, Bangladesh	23.78° N, 90.41° E	DRG	60,662,638	59,298,504	390,962	62.04	756	SRR32835631
3	DR3	DNCC, Bangladesh	23.78° N, 90.41° E	DRG	48,820,994	46,575,074	452,424	61.78	748	SRR32835620
4	DR4	DNCC, Bangladesh	23.78° N, 90.41° E	DRG	44,284,430	43,462,174	513,703	62.94	753	SRR32835612
5	DR6	DNCC, Bangladesh	23.78° N, 90.41° E	DRG	47,117,638	4,622,2048	611,602	63.54	749	SRR32835611
6	DR7	DNCC, Bangladesh	23.78° N, 90.41° E	DRG	49,250,464	48,858,846	1,546,138	44.44	344	SRR32835636
7	DR9	DNCC, Bangladesh	23.78° N, 90.41° E	DRG	44,994,610	44,651,424	2,446,518	46.56	207	SRR32835635
8	DS1	DNCC, Bangladesh	23.78° N, 90.41° E	DSG	51,863,182	51,380,808	2,738,856	42.56	715	SRR32835634
9	DS2	DNCC, Bangladesh	23.78° N, 90.41° E	DSG	43,858,724	43,042,600	839,739	55.96	752	SRR32835633
10	DS3	DNCC, Bangladesh	23.78° N, 90.41° E	DSG	47,000,772	46,186,496	404,485	61.5	746	SRR32835632
11	DS4	DNCC, Bangladesh	23.78° N, 90.41° E	DSG	56,191,782	55,699,876	2,049,273	51.18	730	SRR32835630
12	DS5	DNCC, Bangladesh	23.78° N, 90.41° E	DSG	61,243,454	60,757,998	800,749	42.89	505	SRR32835629
13	DS6	DNCC, Bangladesh	23.78° N, 90.41° E	DSG	42,711,488	42,475,102	1,176,498	40.31	547	SRR32835628
14	GR1	GCC, Bangladesh	23.99° N, 90.38° E	GRG	44,554,198	43,572,430	586,690	60.78	756	SRR32835626
15	GR2	GCC, Bangladesh	23.99° N, 90.38° E	GRG	50,425,924	49,278,262	488,199	62.43	751	SRR32835627
16	GR3	GCC, Bangladesh	23.99° N, 90.38° E	GRG	50,775,720	50,397,022	2,099,824	45.54	691	SRR32835625
17	GR4	GCC, Bangladesh	23.99° N, 90.38° E	GRG	42,067,910	41,566,356	285,853	38.89	432	SRR32835624
18	GR5	GCC, Bangladesh	23.99° N, 90.38° E	GRG	43,335,504	42,779,038	1,919,058	41.78	712	SRR32835623
19	GR6	GCC, Bangladesh	23.99° N, 90.38° E	GRG	43,906,704	43,422,746	640,775	41.71	405	SRR32835622
20	GR7	GCC, Bangladesh	23.99° N, 90.38° E	GRG	45,894,154	45,222,888	208,619	64.29	733	SRR32835621
21	GR9	GCC, Bangladesh	23.99° N, 90.38° E	GRG	44,698,694	44,209,214	2,347,772	44.23	687	SRR32835619
22	GS1	GCC, Bangladesh	23.99° N, 90.38° E	GSG	66,333,510	65,834,502	936,620	39.03	84	SRR32835618
23	GS2	GCC, Bangladesh	23.99° N, 90.38° E	GSG	60,495,574	59,960,662	1,510,564	42.49	409	SRR32835617
24	GS3	GCC, Bangladesh	23.99° N, 90.38° E	GSG	50,210,436	49,409,548	348,061	60.37	756	SRR32835615
25	GS4	GCC, Bangladesh	23.99° N, 90.38° E	GSG	46,783,876	45,616,480	240,224	63.87	723	SRR32835616
26	GS5	GCC, Bangladesh	23.99° N, 90.38° E	GSG	47,019,134	46,116,762	500,388	59.88	746	SRR32835614
27	GS7	GCC, Bangladesh	23.99° N, 90.38° E	GSG	50,644,536	49,551,752	332,346	61.08	755	SRR32835613

^*a*^ DNCC, Dhaka North City Corporation; GCC, Gazipur City Corporation; DR, Dhaka rooftop; DS, Dhaka surface; GR, Gazipur rooftop; GS, Gazipur surface.

By employing WMS, this study presents a comprehensive catalog of microbiome diversity and composition in rooftop and surface garden soils from Bangladesh, offering insights into the role of microbial communities in urban agriculture. WMS of 27 soil samples from rooftop and surface gardens in Dhaka and Gazipur yielded 1.34 billion reads (avg. 49.8 M/sample), with 27.8 M high-quality reads (2.1% of total) mapped to 766 operational taxonomic units (Table 1). A total of 19 phyla, 31 classes, 75 orders, 122 families, 275 genera, and 755 species were identified (https://doi.org/10.6084/m9.figshare.28826870.v1). Firmicutes (65.3%–83.2%) and Proteobacteria (3.4%–24.9%) were dominant, with *Bacillales* (59%–62%) as the leading order. Genus *Bacillus* was highly abundant (>54%) across all metagenomes. DRG showed high *Bacillus paralicheniformis* (28.3%) and *Bacillus licheniformis* (25.2%), while DSG exhibited greater diversity, with dominant species including *Brevibacillus agri* (12.1%) and *Flavobacterium thermophilum* (11.4%). GSG uniquely hosted *Rhodanobacter lindaniclasticus* and *Acidovorax kalamii*. In conclusion, this study highlights *Bacillus* species as prevalent microbes in urban garden soils, serving as a resource for future microbiome research to enhance soil health and agricultural sustainability.

## Data Availability

The whole metagenome shotgun sequencing data are available at the NCBI Sequence Read Archive (SRA) under BioProject accession number PRJNA1044904. The accession numbers for all 27 SRA experiments are listed in Table 1. Taxonomic information is available at https://doi.org/10.6084/m9.figshare.28826870.v1.
